# Supplementary material for: Extraordinary wavelength reduction in terahertz graphene-cladded photonic crystal slabs
Source: Sci Rep. 2016 May 4;6:25301. doi: 10.1038/srep25301 (PMC4855219; doi:10.1038/srep25301)
Supplement: Supplementary Information [file srep25301-s1.pdf]

Extraordinary wavelength reduction in terahertz graphene-cladded photonic crystal slabs

Ian A. D. Williamson[1], S. Hossein Mousavi[1], Zheng Wang[1,*]
[1]Microelectronics Research Center, Department of Electrical and Computer Engineering, The University of Texas at Austin, Austin, TX 78758 US
[*] Corresponding author: zheng.wang@austin.utexas.edu

Photonic band structure without graphene

In Fig. S1(a) we show the bands of the 2D photonic crystal with the band gap range shaded in grey. This band structure is nearly identical to Fig. 1(c), aside from the significant scaling down in frequency that results from graphene's large kinetic inductance.

Fig. S1(b) and Fig. S1(c) show the bands of the dielectric photonic crystal slab for two thicknesses *without graphene*. We observe that no photonic band gap exists and, in the thinner slab, the modes are only weakly guided.

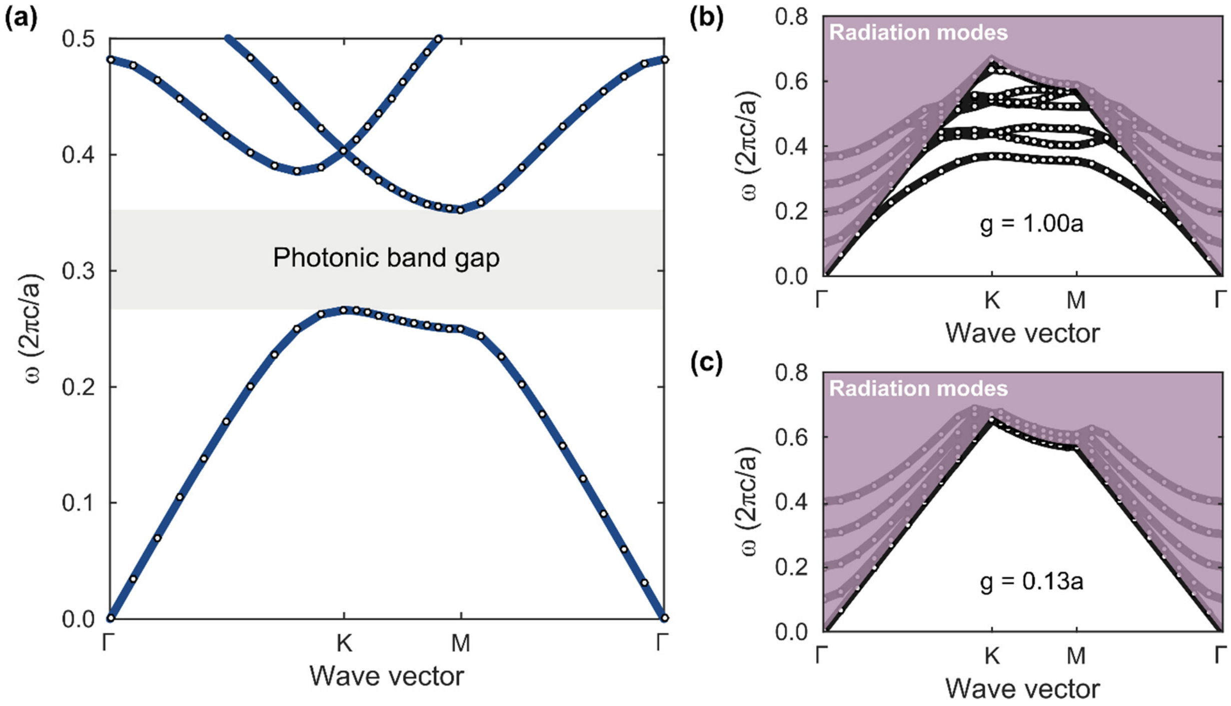

Figure S1 – (a) Band structure of purely 2D crystal having same geometric and material parameters as Figure 1 in main text, computed with MPB. (b),(c) Band structure of finite slab equivalent with thickness of 1.00a and 0.13a. The radiation modes of the substrate and superstrate limit the maximum frequency of the band gap and cause the photonic band gap to be incomplete.

Higher order graphene plasmonic mode dispersion

In Fig. S2 we plot the dispersion of the graphene plasmonic mode with anti-symmetry electrical field distribution along the out-of-plane direction(red dashed line). This odd plasmonic mode is much closer to the light line and is not well confined to the graphene photonic crystal slab, having a significant evanescent tail that extends above and below the slab.

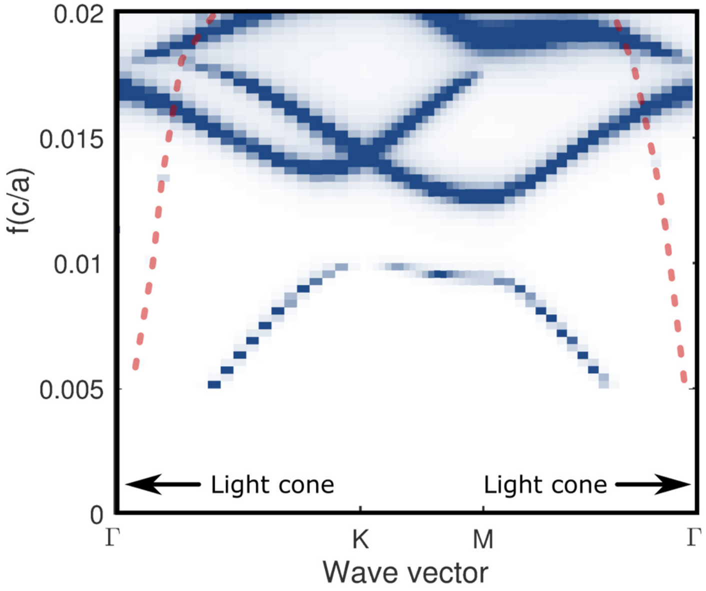

Figure S2 – Energy coupled into guided modes from a small dipole (blue color intensity). The light cone is compressed against the left and right vertical axes. The red dashed line traces the faint band corresponding to the odd graphene plasmonic mode.
